# Molecular prevalence, associated risk factors and phylogenetic evaluation of *Theileria lestoquardi* in the blood samples of small ruminants

**DOI:** 10.1371/journal.pone.0306697

**Published:** 2024-07-11

**Authors:** Sehrish Ashraf, Wafaa M. Hikal, Ruoa Almahallawi, Hira Muqaddas, Furhan Iqbal

**Affiliations:** 1 Department of Zoology, Emerson University Multan, Multan, Pakistan; 2 Department of Zoology, Ghazi University Dera Ghazi Khan, Ghazi Khan, Pakistan; 3 Department of Biology, Faculty of Science, University of Tabuk, Tabuk, Saudi Arabia; 4 Department of Biology, University College of Duba, University of Tabuk, Tabuk, Saudi Arabia; 5 Department of Zoology, The Women University Multan, Multan, Pakistan; 6 Institute of Zoology, Bahauddin Zakariya University Multan, Multan, Pakistan; Benha University Faculty of Veterinary Medicine, EGYPT

## Abstract

Raising small ruminants is the main source of income for farmers in Pakistan especially in rural areas of Dera Ghazi Khan in Punjab. Despite having large sheep population, the prevalence of intra-erythrocytic protozoa, *Theileria* (*T*.) *lestoquardi*, has never been reported from this area. This study was conducted to fill this knowledge gap and 333 blood samples of apparently healthy small ruminants (168 sheep and 165 goats) along with their epidemiological data were collected from Dera Ghazi Khan district during August till November 2022. The polymerase chain reaction (PCR) analysis amplified a 785 base pair amplicon specific for the Merozoite surface antigen (*ms 1–2*) gene of *T*. *lestoquardi* in 2 out of the 168 (3.3%) sheep blood samples, while no goat blood sample out of 165 was found to be infected with *T*. *lestoquardi*. DNA sequencing confirmed the presence of *Theileria lestoquardi* in both samples and phylogenetic analysis revealed that these amplicon resembled the partial *ms 1–2* gene sequences detected in small ruminants from Pakistan, India Iran and Egypt. All the studied epidemiological factors (age, sex, breed, size of herd, dogs with herd, composition of herd, size of herd and Tick burden on sheep) were not found associated with the prevalence of *T*. *lestoquardi*. In conclusion, this study reports a low prevalence of *T*. *lestoquardi* infection in the Dera Ghazi Khan District of Punjab, Pakistan. The data generated from this work will help pave the way for the prophylactic detection and control of ovine and caprine theileriosis in the region.

## Introduction

Pakistan, predominantly an agricultural country, relies heavily on its agriculture sector, with livestock contributing significantly to the economy. In the financial year 2022–23, livestock accounted for 63% of the agricultural revenue through the production of milk, meat, skin, and related products [1]. The country boasts 78.2 million goats (25 breeds) and 30.9 million sheep (24 breeds), primarily located in mountainous regions [2]. Small ruminants like goats and sheep are the livestock of choice for many Pakistani small farm owners. Because they develop quickly, are economically viable, and require less labour-intensive farming [3]. Breeds of sheep can be categorized as thin-tailed or fat-tailed (Balkhi, Balochi, Bibrik, Dumbi, Gojal, Harnai, Hashtnagri, Khijloo, Kohai Ghizar, Latti, Michni, Rakhshani, Tirahi and Waziri). While Beetal, Dera Din Pannah, Kamori, Nachi, Teddy, Dessi, Lail puri and Roucher are among the popular goat breeds in Pakistan [4]. Despite the importance of small ruminants to the rural economy the production by these animals is not as efficient as it should be [5]. Due to the subtropical conditions in Pakistan, ticks and associated tick-borne diseases are abundant in domesticated animals, especially in the arid regions of Punjab with higher temperatures and humidity during most part pf the year [6]. Ticks and their transmitted pathogens cause weight loss, skin damage, mortality and abortion resulting in huge annual economic losses [7]. It has been estimated that the global production economic losses caused by ticks and tick borne diseases are 14–19 billion USD per year and tropical and sub-tropical reagions of the world bear the most out of it [2].

*Theileria lestoquardi* is an obligate intra-erythrocytic protozoan parasite that is known to be transmitted to the small ruminants through a number of tick vectors including *Rhipicephalus turanicus*, *Hyalomma* (*H*.) *anatolicum anatolicum*, *H*. *detritum*, *H*. *impeltatum* and *H*. *excavatum* and cause theileriosis in them [8]. *T*. *lestoquardi* infection is currently regarded as one of the greatest obstacles to the sheep and goat production as the parasite infect lymphocytes and causes mortality due to significant tissue damage and pulmonary edema that eventually results in respiratory failure [9]. The morbidity rate in small ruminants infected with *T*. *lestoquardi* can approach 100% with reported mortality rates of 46–100%. The sickness is characterized by fever, weakness, anorexia, concurrent petechiae, swollen lymph nodes, anaemia and cough [10].

District Dera Ghazi has no industries or alternative ways of earning and the local population is exclusively depends upon agriculture and livestock for their bread and butter [1]. It is a very common practice of the local population to rear sheep and goats in order to meet the growing of mutton as mutton is the meat of choice among Pakistani population [2]. Due to its varied ecosystem, animals of this region are vulnerable to tick infestation. Although a recent study conducted in this area has reported the presences of *Theileria ovis*, *Anaplasma ovis* and *Anaplasma marginale* (all tick borne pathogens) in goats and sheep [1]. Still there was no report regarding the presence of *Theileria lestoquardi* in small ruminants of this area despite the fact that *Theileria lestoquardi* infection has high rates or morbidity and mortality among sheep and goats. The present study was designed to investigate the molecular prevalence of *T*. *lestoquardi* in blood samples collected from sheep and goats across different localities in Dera Ghazi Khan. Additionally, the study aimed to report on the genetic diversity of the parasite and identify any risk factors associated with this infection.

## Materials and methods

### Study area, blood and data collection

**Fig 1:**
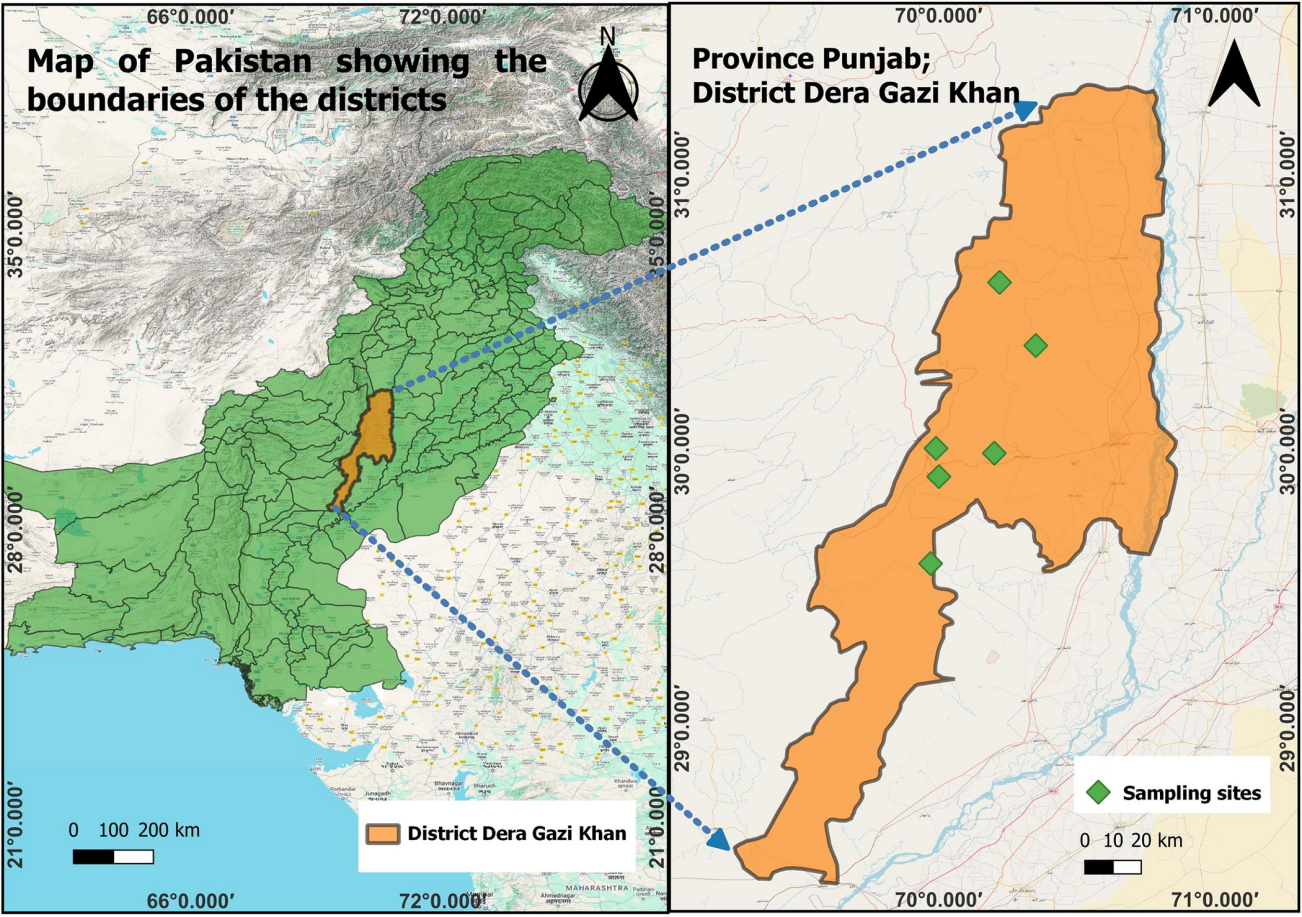
Map of Pakistan with highlighted Dera Ghazi Khan district. Magnified map is showing the sites from where the small ruminant samples were collected during present study in Dera Ghazi Khan district.

Ethical Research Committee of the Department of Zoology at Ghazi University Dera Ghazi Khan (Pakistan) approved all the experimental procedures and protocols applied in this study via letter number Zool./ Ethics/22-18. District Dera Ghazi Khan is located at 30° 1’ 59" N, 70° 38’ 24" E. It is located in a strip between the river Indus and the Koh-Suleman range of mountains separating it from the Baluchistan Province. The overall climate of this district is dry with very little rainfall. The winter is mild, lasting from November till January with average temperature 4°C but summer is extremely hot and extended, starting in May and lasting till September with average temperature of 42°C [7]. Following informed oral consent of livestock owners, a total of 333 small ruminants, including apparently healthy sheep (N = 168) and goats (N = 165), were enrolled from various areas in Dera Ghazi Khan District of Punjab. The sampling sites included Anari Top, Lukii, Gagan Thal, Chitri, Neelani and Shum (Fig 1). A questionnaire was filled on the sampling site in order to collect data regarding epidemiological factors (age, sex, breed, size of herd, dogs with herd, composition of herd, size of herd and tick burden on sheep) associated with the prevalence of *Theileria lestoquardi*. From the jugular vein of each animal, 3–5 ml of blood was collected aseptically by using disposable syringes into an EDTA containing tube and that was later on used for the DNA extraction.

### DNA extraction

DNA was extracted from the collected blood samples using an inorganic method which involves temperature and enzyme shock. Briefly, blood samples were suspended in 500 μL of lysis buffer (20 mM Tris-HCl, 1 mM EDTA, 30 mM DTT, 0.5% SDS) supplemented with 0.4 mg/ mL proteinase K (Fermentas, USA). The samples were kept overnight in a heating block set at 55°C. After the lysis, samples were heated at 95°C for 10 min. Then equal volume of phenol: chloroform: isoamylalcohol mixture (25:24:1 v/v/v) was added, briefly vortexed and centrifuged at 12000×g for 10 min. The aqueous phase was transferred to a new clean tube and equal volume of ice cold isopropanol was added. The DNA was pelleted by centrifugation at 12000×g for 15 min. The pellet washed with 70% ethanol and dried at 65°C for 5 min. The DNA was finally re-suspended in 50 μL sterile distilled water.

### PCR amplification

A pair of oligonucleotides primers, Fwd 5′-GTGCCGCAAGTGAGTCA-3′ and Rev 5′-GGACTGATGAGAAGACGATGAG-3′ was used to amplify a 785 bp fragment specific for the Merozoite surface antigen (*ms 1–2*) gene of *Theileria lestoquardi* following Taha et al. [11]. PCR was carried out in a 25 μl reaction mixture containing 10X buffer A [500 mMKCl, 100 mMTris-HCl] (pH 9.1), 250 ng genomic DNA, 12 pM of each primer, 0.16 mM of dNTPs, 2.5 U of Taq DNA polymerase (Vivantis, UK) and 1.5 mM MgCl_2_ [11]. Thermo profile comprised of an initial denaturation step at 94°C for 5 min followed by 30 cycles of denaturation at 95°C for 50s, primer annealing at 59°C for 50 s and extension at 72°C for 50s. A final extension at 72°C for 5 min was also performed [11]. Positive controls (previously sequence confirmed *T*. *lestoquardi* DNA isolated from sheep, accession number MZ541895) was used in PCRs and double distilled water served as negative control during each reaction.

### DNA sequencing and phylogenetic analysis

Amplified parasite positive PCR products were sequenced from a commercial lab (First Base, Malaysia). The obtained DNA sequences underwent initial trimming to eliminate primer-contaminated regions and any misread nucleotides at the start and end of the sequence were also removed using FinchTV (version 1.4.0). Both sequences were submitted to NCBI’s GenBank and were assigned accession numbers: PP719440 and PP719441. Additional similar sequences were retrieved using the Basic Local Alignment Search Tool (BLAST) algorithm on NCBI’s platform. Subsequently, all the sequences were aligned using the ClustalW multiple sequence alignment algorithm in BioEdit (version 7.2.5). The aligned sequences were imported into MEGA X (version 10.2.6). A model selection test was conducted for all sequences, using MEGA’s integrated model selection tool. The best-fit model was chosen based on Bayesian Information Criteria (BIC) and Akaike Information Criteria (AIC) values, with the model exhibiting the lowest BIC and AIC considered as the "best-fit" substitution model. Phylogenetic trees were constructed using the Maximum Likelihood algorithm in MEGA X with 1000 bootstraps. The final version of the inferred tree was generated using the iTOL server (https://itol.embl.de/, accessed on April 24^th^ 2024).

### Statistical analysis

Data analysis was conducted using the Statistical Package (Minitab 21, USA). One-way analysis of variance (ANOVA) was used to compare the prevalence of *T*. *lestoquardi* among different sheep and goat breeds and sampling sites. Epidemiological data were correlated with the presence of *T*. *lestoquardi* by using the Fischer’s exact test.

## Results

### Prevalence of *Theileria lestoquardi* in blood samples of sheep and goat

Polymerase chain reaction amplified a 785 base pair amplicon specific for Merozoite surface antigen (*ms 1–2*) gene of *T*. *lestoquardi* in 2 out of 168 (3.3%) sheep blood samples collected from district Dera Ghazi Khan during present study, while no goat blood sample was found infected with *Theileria lestoquardi*.

### Phylogenetic analysis of *Theileria lestoquardi* based on Merozoite surface antigen *(ms 1–2)* gene

**Fig 2:**
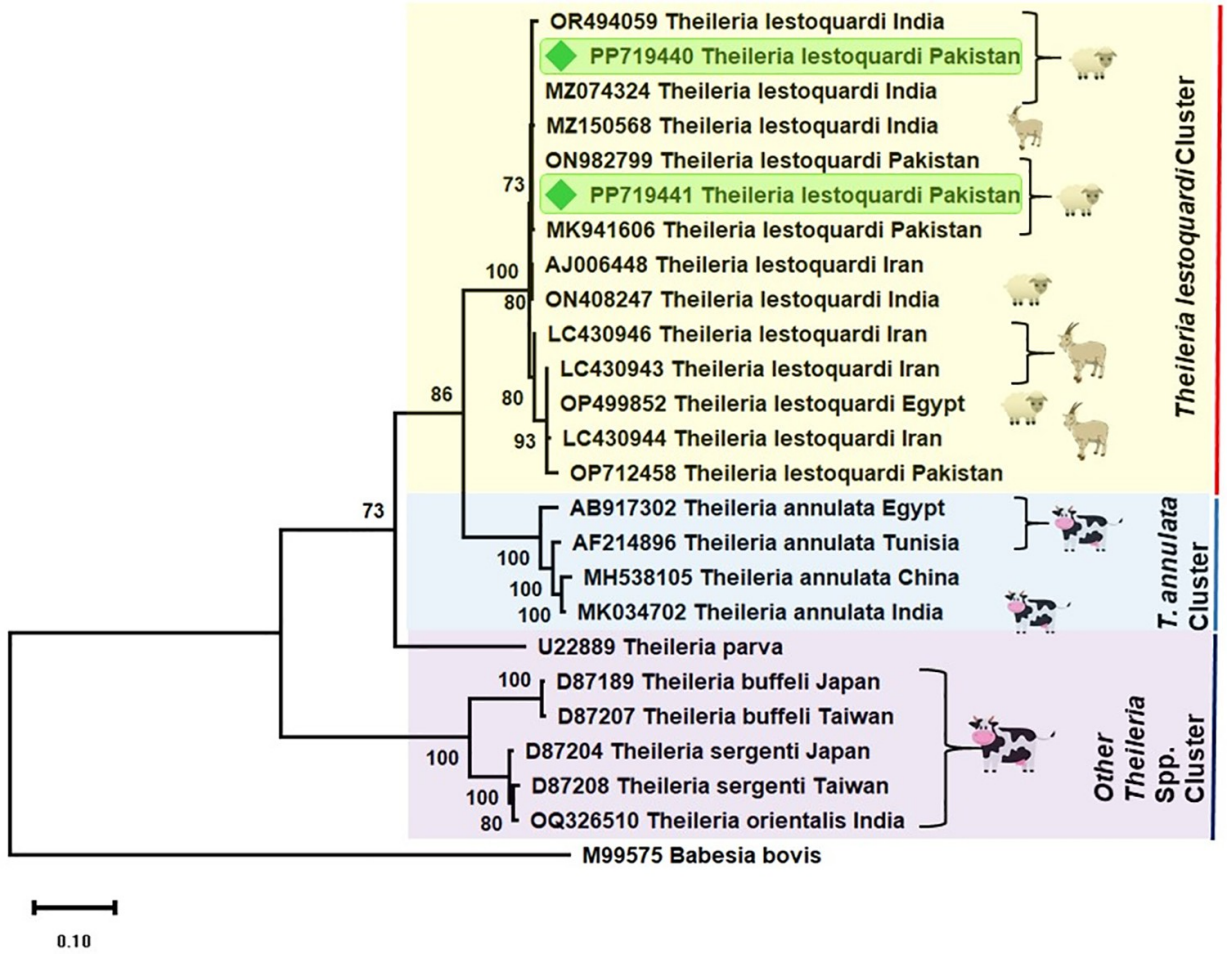
Phylogenetic tree of *Theileria lestoquardi* based on the partial Merozoite surface antigen (*ms 1–2*) gene sequences. The two new sequences of *Theileria ovis* obtained in this study are highlighted with red square. Scale bar represents 0.10 substitutions per nucleotide position.

A total of two *T*. *lestoquardi* isolates were sequenced and only one genotype was identified after the alignment of *ms 1–2* partial nucleotide sequences (735 bp). These two isolates were submitted in GenBank under accession numbers PP719440 and PP719441, respectively. BLAST analysis revealed 99–100% sequence homology of our amplified DNA sequences with the partial *ms 1–2* sequences available in GenBank. Phylogenetic analysis showed that our isolates were clustered in the same clad with those previously reported from Pakistan (ON982799, MK941606, OP712458), India (OR494059, MZ074324, MZ150568, ON408247), Iran (LC430943, LC430944, LC430946, AJ006448) and Egypt (OP499852) (Fig 2).

### Risk factor analysis

When prevalence of *T*. *lestoquardi* was compared between enrolled sheep, one way ANOVA results revealed that during present investigation, parasite prevalence was not restricted to a particular sampling site (P = 0.653) or breed (P = 0.655) (Tables 1 and 2). Risk factor analysis revealed that none of the studied parameter was found associated with the prevalence of *T*. *lestoquardi* in sheep during present study (P > 0.05) (Table 3).

**Table 1:** Prevalence of *Theileria lestoquardi* among various sheep breeds enrolled from district Dera Ghazi Khan.

Sheep breeds	N	Positive (Rate, %±C.I.^1^)	P-value
Baluchi Dumba	76	2 (2.63±0.035)	
Baluchi Dumbi	50	0 (0)	
Waziri Dumba	08	0 (0)	
Awassai sheep	07	0 (0)	
Balkhi Sheep	06	0 (0)	
Latti	06	0 (0)	0.653
Afghan Arbi	04	0 (0)	
Chakki Dumba	04	0 (0)	
Deccani	03	0 (0)	
Damani sheep	01	0 (0)	
Bibrik	01	0 (0)	
Assaf	02	0 (0)	
Total	**168**	2 (1.19±0.015)	

^1^ C.I.: 95% confidence interval, P > 0.05 = Non signifiant

**Table 2:** Association of *Theileria lestoquardi* prevalence in various sheep breeds enrolled during present study from district Dera Ghazi Khan.

Sampling sites	Total	Positive (Rate, %±C.I.^1^)	P-value
Anari top	23	0 (0)	0.655
Lukki	27	0 (0)	
Chitri	33	1 (3±0.058)	
Neelani Shum	40	0 (0)	
Gagan Thal	45	1 (2±0.043)	
Total	168	2 (1.19±0.015)	

^1^ C.I.: 95% confidence interval, P > 0.05 = Non significant

**Table 3:** Association of *Theileria lestoquardi* prevalence with the studied epidemiological parameters describing sheep character enrolled during the present study from district Dera Ghazi Khan. N represents the total number of collected samples. % prevalence of *Theileria lestoquardi* is given in parenthesis. P-value represents the results of Fischer Exact test calculated for each studied parameter.

Factors	Classes	Total	Positive (Rate, %±C.I.^1^)	P-value
Sex	Male	25	1 (4±0.076)	0.161
	Female	143	1 (0.70±0.013)	
Age	≤ 1 year	18	0 (0)	0.623
	> 1 year	150	2 (1.33±0.017)	
Composition of herd	Sheep only	45	0 (0)	0.390
	Sheep and goats	123	2 (1.62±0.021)	
Dogs with herd	Present	77	1 (1.29±0.025)	0.905
	Absent	91	1 (1.09±0.021)	
Size of herd	≤ 30	24	1 (4.16±0.080)	0.942
	> 30	43	1 (2.32±0.045)	
Tick burden on sheep	Yes	92	1 (1.08±0.021)	0.892
	No	76	1 (1.31±0.025)	

^1^ C.I.: 95% confidence interval, P > 0.05 = Non significant

## Discussion

Tick-borne protozoal diseases are economically significant in Pakistan, as they can cause serious health issues that impair animal productivity [12–14]. To develop and implement effective control strategies, epidemiological data on these infections and their impacts is necessary [15]. The present study was conducted to investigate the prevalence and genetic diversity of the highly virulent *T*. *lestoquardi* parasite in blood samples collected from goats and sheep in Dera Ghazi Khan, Punjab, Pakistan.

During the present investigation, none of the screened goats was found to be infected with *T*. *lestoquardi*. However, the ms 1–2 gene of *T*. *lestoquardi* was amplified in 3.3% of the sheep blood samples (Table 1). Previous studies from Pakistan have reported varying prevalence rates of this parasite in different regions, particularly within the Punjab province. A recent study by Tanveer et al. [10] found a very low prevalence (1.2%) of *T*. *lestoquardi* in sheep from Rajanpur District, Punjab. Similarly, Fatima et al. [16] reported a 3.47% PCR-based prevalence of the parasite in apparently healthy small ruminants across five districts in Southern Punjab, which is comparable to the findings of the present investigation. In contrast, some other studies have reported higher prevalence rates, such as 35.2% in small ruminants from Multan District [17] and 26.2% in sheep from Baluchistan [18]. Durrani et al. [19] also found that 22% of small ruminants in Lahore District were microscopically positive for *Theileria* spp., although PCR detected ovine theileriosis in only 35% of these samples, highlighting the higher sensitivity and reliability of molecular techniques. The differences in prevalence rates across these studies may be attributed to various factors, including geographical location, climate, tick density, host age, gender, immunity, and management practices [20].

While there are few reports available on the genetic diversity of *T*. *lestoquardi* in Pakistan based on the parasite’s partial merozoite surface antigen (ms 1–2) gene sequences from small ruminants, this information has been limited to selective sampling sites. Notably, the genetic diversity of *T*. *lestoquardi* in goats from the Muzaffargarh district has not been previously reported. The present investigation aimed to address this knowledge gap. BLAST analysis of the partial sequences confirmed that the goats were infected with *T*. *lestoquardi*, and the phylogenetic study revealed that the DNA sequences amplified during this investigation were genetically similar to *T*. *lestoquardi* sequences from sheep and goats in Pakistan (Accession numbers ON982799, MK941606, and OP712458, *unpublished*), small ruminants in India (Accession numbers OR494059, MZ074324, MZ150568, and ON408247, *unpublished*), goats in Iran (Accession numbers LC430943, LC430944, and LC430946, [21]), and small ruminants in Egypt (Accession number OP499852, unpublished). This limited and largely unpublished data suggests a need for more extensive studies reporting the prevalence and genetic diversity of *T*. *lestoquardi* in small ruminant species across different regions of Pakistan and the world. Such studies would contribute to a better understanding of the genetic diversity of this important protozoan parasite, which is crucial for designing effective control strategies and reducing economic losses.

The risk factor analysis revealed that the prevalence of *T*. *lestoquardi* was not restricted to any particular sheep breed, sampling site, age, or sex. Additionally, the parasite prevalence was not associated with herd composition, the presence of ectoparasites on the animals, or the association with dogs within the herds (Tables 1–3). No such information can be provided for goats as we did not detect this parasite among the screened goats during present investigation. The apparent reason that we did not find any risk factor associated with *T*. *lestoquardi* infection was the low parasite prevalence observed during present investigation. This data is indicating that small ruminants of the studied areas have developed immunity against one of the most fatal piroplasm: *Theileria lestoquardi*.

The findings of the present study align with previous research on *T*. *lestoquardi* in small ruminants in Pakistan. Riaz and Tasawar [17] also reported that *T*. *lestoquardi* infection was not restricted to any particular small ruminant breed in their study from Multan district. Similarly, Naz et al. [22] and Saeed et al. [23] found no association between age, sex, or sampling season and theileriosis in sheep and goats from Lahore. Shahzad et al. [24] likewise determined that sheep breed had no significant association with *Theileria* spp. infections. Furthermore, Tanveer et al. [10] did not observe any association between *T*. *lestoquardi* prevalence and the risk factors examined in small ruminants from Southern Punjab. There are some studies that have reported results that are contrary to our findings. Iqbal et al. [25] had reported higher parasite prevalence in males while Naz et al. [22] found this infection more prevalent in female than male.

In conclusion, we are reporting a relatively lower prevalence of *T*. *lestoquardi* among the enrolled sheep from Dera Ghazi Khan District. The parasite prevalence was not associated with any of the studied risk factors. While we have 333 blood samples of small ruminants during present study, we consider it a small sample number and suggest that studies enrolling higher number of animals should be conducted to have a better understanding for *T*. *lestoquardi* infection status among local small ruminants. Awareness regarding ticks and their control must be provided to livestock owners in Punjab to further reduce the incidence of tick borne diseases. The PCR protocol used in this study can be used for effective prophylactic detection of this pathogen among local small ruminants that will lead towards the control of tick borne diseases among Pakistani livestock.
